# South African primary health care allied health clinical practice guidelines: the big picture

**DOI:** 10.1186/s12913-018-2837-z

**Published:** 2018-01-29

**Authors:** J. M. Dizon, K. A. Grimmer, S. Machingaidze, Q. A. Louw, H. Parker

**Affiliations:** 1 International Centre for Allied Health Evidence, University of South Australia, Adelaide, 5000 Australia; 2 Centre for Evidence-Based Health Care (CEBHC), Faculty of Medicine and Health Sciences, Stellenbosch University, Francie van Zijl Drive, Tygerberg, Cape Town, 7505 South Africa; 3 Department of Physiotherapy, Faculty of Medicine and Health Sciences, Stellenbosch University, Francie van Zijl Drive, Tygerberg, Cape Town, 7505 South Africa; 4 Cochrane South Africa, South African Medical Research Council, Francie van Zijl Drive, Parow Valley, Cape Town, 7505 South Africa

## Abstract

**Background:**

Good quality clinical practice guidelines (CPGs) are a vehicle to implementing evidence into allied health (AH) care. This paper reports on the current ‘state of play’ of CPGs in a lower-to-middle-income country (South Africa), where primary healthcare (PHC) AH activities face significant challenges in terms of ensuring quality service delivery in the face of huge PHC need.

**Methods:**

A qualitative study was conducted, using semi-structured interviews with purposively-sampled individuals involved in AH PHC CPGs in South Africa. They included national and state government policy-makers, academics and educators, service managers, clinicians, representatives of professional associations, technical writers, and members of informal professional networks. The interview data was transcribed and de-identified, and analysed descriptively by hand-coding. The COREQ statement guided study conduct and reporting. A framework to guide research in other countries into perspectives of AH PHC CPG activities was established.

**Results:**

Of the 32 invited, 29 people participated: of these 25 were interviewed and four provided meeting notes. Most participants had multiple professional roles, being engaged concurrently in clinical practice, academia, professional associations and / or government. Key themes comprised Players (sub-themes of sampling frame, participants, advice, role players and collaboration); Guidance (sub-themes of nomenclature, drivers, purpose, evidence sources) and Role of AH in PHC (sub-themes of discipline groupings, disability and rehabilitation, AH recognition).

**Conclusion:**

There was consistently-expressed desire for quality guidance to support better quality AH PHC activities around the country. However no international CPGs were used, and there were no South African CPGs specific to local PHC AH practice. The guidance gap was filled by non-evidence-based documents produced often without training, to deal with specific clinical situations. This led to frustration, duplication and fragmentation of effort, confusing nomenclature, and an urgent need for standardised and agreed guidance. We provided a standardised framework to capture perspectives on CPGs activities in other AH PHC settings.

**Electronic supplementary material:**

The online version of this article (10.1186/s12913-018-2837-z) contains supplementary material, which is available to authorized users.

## Background

Clinical Practice Guidelines (CPGs) were defined by the United States of America Institute of Medicine in 2011 as *‘statements that include recommendations intended to optimize patient care that are informed by a systematic review of evidence and an assessment of the benefits and harms of alternative care options’* (p.4) [1]. Recently they were described as ‘*a convenient way of packaging evidence and presenting recommendations to healthcare decision makers*‘(p.6) [2].

Methodologically-sound CPGs should be developed using standard internationally-agreed principles, and be based on the best current evidence [3]. Such CPGs can provide policy-makers, clinicians, funders and service managers with time-efficient and reliable sources of current evidence on which to base service-delivery decisions [4]. CPGs may also include ‘how to do it’ documents such as written recommendations with underpinning levels of evidence, algorithms and patient decision/ management tools, protocols or directives [5]. These are designed for busy end-users (clinicians, managers) who require readily accessible information about putting best evidence into practice [3, 4].

CPGs usually deal with one or more questions about screening, diagnosis, treatment, monitoring and/or cost-effectiveness of care decisions [6]. CPGs which include allied health (AH) recommendations for specific conditions are increasingly available internationally. AH is an umbrella term, encompassing health disciplines which are often defined by exclusion (not medicine, nursing, veterinary science or dentistry), and where AH professions are variably named in lists, depending on where they work [7–9]. Generally AH care focuses on morbidity, not mortality [9]. It is currently accepted that ‘AH’ encompasses therapies, diagnostic and technical, scientific and complementary disciplines/professions [7–9]. AH therapies, the focus of the research reported in this paper, provide screening, diagnosis, treatment, education and /or monitoring services across the lifespan, mostly to alleviate sequelae of disease [7–9]. AH therapies provide care for individuals in primary, secondary and tertiary settings, to improve their function and quality of life from injury or illness [8, 9].

The Guidelines Intercollegiate Network (G-I-N) recently produced a position statement outlining four themes (comprising 14 items) relevant to enhancing a person-centred approach in AH CPGs *(‘i. use a joint definition of health-related quality of life as an essential component of intervention goals, (ii) incorporate the International Classification of Functioning, Disability and Health (ICF) as a framework for considering all domains related to health, (iii) adopt a shared decision-making method, and (iv) incorporate patient-reported health outcome measures’* (p 1543) [10]. Often, AH recommendations are embedded in condition-specific CPGs which provide guidance to other healthcare providers (e.g. Australian Guidelines on Management of Type 2 Diabetes 2011 [11]). Thus AH providers interested in using CPGs may have to search first for a condition-specific CPG and then find discipline-relevant recommendation(s) embedded within it. Whether AH recommendations are provided in a disease-specific CPG depends on CPG purpose and intended end-users. To improve ease of access for end-users, profession-specific summary guidance can also be produced from a large multidisciplinary CPGs (e.g. separate guidance documents for physiotherapy, occupational therapy, speech and language/hearing and social work produced from the Australian National Stroke Foundation CPG for acute stroke management [12]. Profession-specific CPGs are also available, usually produced by professional associations (eg American Physical Therapy Association low back pain CPG) [13]. Access to, and uptake of, AH CPGs is aided by freely-available ‘one-stop-shops’ such as CPG repositories on respected CPG developers’ websites (eg Scottish Intercollegiate Guidelines Network website (http://www.sign.ac.uk/)) [14] or national independent CPG clearing houses (such as hosted by US Agency for Healthcare Research and Quality http://www.guideline.gov/) [15].

Effectively implementing CPGs into every-day AH practice is an evolving area of research [10, 16, 17]. Whilst good quality CPGs are generally available at no cost for many health conditions, barriers to uptake of CPGs by AH providers have been consistently reported. These include lack of time and knowledge to find CPGs, constrained knowledge about CPG construction and how to assess their quality, lack of organisational will and/or managerial support to embed CPGs routinely into local practice, limited access to CPGs because of infrastructure constraints (eg internet access or library facilities), and local referral and/or prescription systems which may restrict AH clinical autonomy [15–19].

The World Health Organisation (WHO) reported on characteristics for good quality service delivery (WHO 2010), which need to be considered in AH CPGs [20]. This work importantly separates best practice interventions (mostly derived from experimental studies) from operationalisation of services (how best to put interventions into practice). Quality service delivery characteristics reflect inputs (Workforce; Service comprehensiveness; Resources; Continuity; Coordination; Accountability) and outputs (quality care processes, and quality health outcomes). Outputs can be measured in different ways including Person-centredness; Efficiency; Equality (individual rights to care); Equity (coverage); Access; Timeliness; and Effectiveness. Provision of quality service delivery guidance in CPGs used by AH therapists, managers and policy-makers is as important as information on interventions, because by its very nature, quality AH care is not just about what is done, but also about who does it, how often it is done, how much care is delivered, who takes responsibility for it and how it integrates with patient-locus of control [7–9, 15–19, 21].

Internationally there is a lack of information on if, how,and why CPGs are used in AH, and whether the benefits of using them outweigh the costs [8–10, 22, 23]. This paper reports on perspectives of CPGs by end-users in primary health care (PHC) settings in South Africa (SA). Resources for AH generally, and for any AH CPG activity, have been constrained for three decades in SA, despite an escalating and currently unmet need for best-evidenced, standardised guidance to address the increasing prevalence of communicable and non-communicable diseases [24–26]. Since it became a government priority in the 1994 National Health Plan, SA has consistently acknowledged the importance of PHC [27, 28]. PHC providers (GPs, nurses or allied health practitioners) attend to South Africans’ healthcare needs over their lifespan, and PHC is usually the point of patient entry into the SA healthcare system [24, 25]. In SA, clinical guidance is developed by many groups including the National and Provincial Departments of Health, and professional societies [28]. There is however, no central, nationally recognized and accepted CPG development unit in place, and no support for AH CPG activities to improve PHC quality and health outcomes.

Moreover, whilst there is a regulatory authority (Allied Health Professions Council of South Africa) [29], there is little publically-available information on what any AH discipline does, to whom or how, or with what outcome. SA is a land of contrasts, evident in variable AH care provision, access, affordability and consumption. Some patients can access the very latest technology in large tertiary hospitals, whilst others can only access basic levels of PHC in rural communities [28]. Geography, culture, economy, education and social justice play a large part in who consumes SA AH services, and with what outcome. Thus people’s capacity to pay, their belief in the benefits of different AH providers, and providers’ skill sets and availability, will often dictate patient choice in consuming treatment [22, 25, 26, 28]. In the past 20 years, there has been little SA focus on the importance or benefit of AH PHC care, as government priorities have been on reducing mortality from communicable diseases such as HIV/AIDS, TB and malaria [28–31]. Now that this war is gradually being won, and more people are now living with these diseases as chronic conditions, recognition is growing of AH as having the requisite skills to optimise function and quality of lives saved [29–32]. Anecdotally, the demand for PHC AH services is escalating, but this is not currently matched by sufficient workforce or resourcing, nor on a sound evidence base for practice [30–32].

This paper reports findings from a sub-study in Goal 1, in the South African Guidelines Evaluation (SAGE) project. Project SAGE was funded by a South African Medical Research Council Flagship research grant 2013–2017 [33]. It had five goals. Goal 1 sought information from key medical and policy participants on PHC CPG needs, development, relevance, uptake and implementation in SA. It became apparent during Goal 1 data collection that the voices of other players in SA PHC settings also needed to be heard. Thus the Goal 1 AH sub-study was undertaken to capture perspectives on AH PHC CPG from knowledgeable SA PHC AH policy-makers, academics, managers and clinicians. Key Project SAGE findings are reported in South African Medical Journal editorials [24–27]. Three journal articles report on South African CPG quality [5, 28, 29], and one reported on a novel conceptual framework was reported of stacking tiers of activity to efficiently underpin production of implementable CPGs for local uptake [34].

Given the immense, increasing challenges in front of SA AH policy-makers, managers and clinicians to deal efficiently with the increasing tsunami of people with chronic diseases who require PHC services to improve function and quality of life, the Project SAGE AH substudy was timely. It provided a rare opportunity to explore AH end-user perspectives on CPGs, which could potentially support delivery of cost effective and efficient services to increasing numbers of patients not previously considered in government estimates^4,10,28–31.^ This paper established the current ‘state of play’ of CPG activities in PHC AH in SA by synthesising perspectives of knowledgeable AH players.

## Methods

### Ethics

Ethical approval was provided by the three participating organisations in Project SAGE: Medical Research Council (EC002–2/2014), Stellenbosch University (N14/02/008) and University of South Australia (0000034923).

### Qualitative research approach

The research was conducted and reported in accordance with the COREQ criteria, the current gold standard for qualitative research [35]. This study took a descriptive, inductive approach to develop a framework that would assist us to efficiently analyse data amassed from the stories of individuals aligned with pre-determined clusters of activity relevant to PHC AH CPGs [36–38]. This sampling framework was essential to ensure a rigorous recruitment strategy, and appropriate classification and analysis of the rich interview data provided by participants. Additional file 1 reports the semi-structured interview guide. These questions were the same as those asked of the medical and policy-maker participants in Project SAGE Goal 1 interviews.

### Research team

The team consisted of three AH researchers with experience in CPG writing and implementation (KAG, JMD, QAL), a public health epidemiologist with systems analysis expertise (SM) and a social scientist (HP). The researchers were all experienced interviewers, with formal training in interview and focus group data collection methods, and qualitative research analysis and reporting.

### Therapy focus and overview

The SAGE AH sub-study focused on the four most common AH therapies in South African PHC: physiotherapy, occupational therapy, podiatry, and speech and language/ hearing. In South Africa, these therapies operate in public and private PHC, in metropolitan, regional and rural settings. These AH clinicians generally have first contact practitioner status in the private sector, where they can provide services without a medical referral. However there is variable first contact status in the public sector, depending on the availability of medical practitioners, the environment and usual local practices. In rural public PHC settings, these AH therapists regularly provide services in South African in the absence of a doctor [31].

### Sample

Our sampling reference frame [33] enabled us to identify and purposively recruit for maximum variation, individuals with experience and knowledge of PHC AH CPG activities, using a cluster recruitment strategy [36]. Pre-establishing the sampling frame was critical to complying with COREQ reporting standards 10 and 11 [35]. This approach was particularly important given how little we knew about the activities in CPGs in South African AH PHC. Sampling clusters comprised:

- AH / rehabilitation portfolios of National and four provincial governments. Provinces were selected on heterogeneity of economics, size, population distribution, and access to tertiary training
- managers of rehabilitation services at district and sub-district level
- public sector discipline-specific clinicians at district, sub-districts and community levels
- professional discipline-specific associations
- private sector discipline-specific clinicians
- special interest groups (multidisciplinary or single discipline)
- tertiary training institutions teaching single discipline programs
- medical aids (health insurance companies) and
- CPG writers/ consultants.

### Sample identification

A combination of chain and maximum variation sample generation process [33, 35, 36]started with key informants being identified within the Project SAGE team. These were researchers or students who were not necessarily interviewed, but who knew of key individuals in the pre-identified clusters. They were approached to participate, and even if they did not agree to interview, they were asked for names of others who might meet the inclusion criteria. This approach assisted us to identify relevant AH clusters in different ways (by discipline, organisation, purpose and dependence (or independence) on other clusters). Purposive sampling continued until all clusters had been populated with consenting interviewees.

### Researchers’ relationship with participants

One researcher (QAL) was known to eight participants, and a second researcher (KAG) was known to four participants (four being common to both QAL and KAG). When inviting participants to join the research, the researchers declared their position (and prior knowledge), and the intent of the research. They clarified ways in which participants’ anonymity would be protected. Prior to interview, all interviewees reviewed the information sheet, signed the consent form and clarified issues and concerns.

### Data collection processes

Data were collected formally by individual interview, or focus groups. Signed consent forms were collected and audio recordings were made. Data was also collected informally in individual interviews or small group meetings, where signed consent forms were collected, but by agreement, only meeting notes were taken (no audiotapes). This occurred when participants wanted to talk ‘off the record’. When participants provided ‘off the record’ information, this was used as background material, and/or for verification of information provided in formal recordings.

The researcher worked in pairs when conducting interviews. Interviewer dyads changed regularly. Having the same people involved in data collection maintained continuity in questioning and note-taking, and supported efficient subsequent data handling and analysis [35–37]. At each interview one interviewer led, and one took field notes. This person contributed additional questions to the interview, if further clarity was required. A ‘reflective’ interviewing technique was used, which summarised, in the researchers’ words, what had been heard in the interview, to which participants were invited to clarify, amend or further expand [35–37]. As relevant, issues raised in one interview were introduced in subsequent interviews so that a cohesive and evolving picture of AH CPG perspectives was developed during the study. Member checking of interview transcripts was offered to all participants.

Once we suspected that we had reached saturation in any cluster (where nothing new was heard since the last interview), one further interview (formal or informal) was conducted with the next participant. If no new information was forthcoming, interviewing in that cluster stopped at that point. However, if new information was found in that interview, further sampling and interviewing occurred until data saturation was achieved [35, 36, 39].

### Analysis approach

Analysis was undertaken using paper copies of transcripts, where answers to the semi-structured interview questions were inferred as ‘chunks’ of meaning, from sentences or paragraphs. Transcript analysis was undertaken independently by all authors then meetings were held to discuss themes and sub-themes identified. All authors agreed on the final themes and sub-themes and collaborated in drafting this paper, and identifying exemplar quotations to support better understanding of the ‘current state of play’. The key themes and sub-themes described who was involved in CPG activities (and in what roles), why, and for what purpose, CPG activities were undertaken, CPG terminology, and drivers for CPG activity.

### Building the framework

A framework was developed to capture key issues, wording of questions which could initiate future discussion on these issues, and ways in which this information could be interpreted. This framework assisted us in reporting our findings, and may also inform future research elsewhere, investigating the same concerns.

## Results

We heard consistent reference by most participants to the enormous need in SA PHC for better AH and rehabilitation practices, greater political recognition of the role of AH particularly in PHC, and better quality guidance to underpin AH practices. One participant expressed this issue comprehensively:

*‘to make them (doctors, government) understand that without rehabilitation the burden of care or in terms of mobility burden is spinning out of control, I mean, the country is at increasingly we've got a quadruple burden of disease, people coming with multiple morbidities and a lot of the ability to care or not care for themselves dependent of whether they have access to rehab and whether those families have the support they need to deal with people who have functional challenges and they get the kind of things that promote their independence and their ability to operate in society and without rehab, their chances are very poor’.*

Three key areas and twelve sub-themes were identified: **Players** (sub-themes of sampling frame, participants, advice, role players and collaboration); **Guidance** (sub-themes of nomenclature, drivers, purpose, evidence sources) and **Role of AH in PHC** (sub-themes of discipline groupings, disability and rehabilitation, AH recognition).

### Players

#### Sampling frame

Additional names were mentioned during interviews, however no new AH clusters were identified from those in our sampling strategy [33]. The sampling approaches allowed us to comprehensively capture “… *the core experiences and central, shared dimensions of a setting or phenomenon*” (p. 235) [39]. The data saturated in each cluster before we completed the planned interviews.

#### Participants

Thirty-two individuals were approached for interview on AH CPG activity in PHC, and three refused, all for reasons of lack of supervisors’ approval. Subsequently there were 29 participants, of whom 25 provided formal interviews and four contributed to informal notes. All but eight of the participants had AH therapy backgrounds. Of those remaining, four had medical qualifications, one had a pharmacy background, one had a psychology background, and two had nutrition backgrounds (which is a stand-alone PHC area in South Africa (not considered an AH therapy)). Most participants wore more than one professional ‘hat’. These are described in Table 1. Participants are listed by an identification number, and the primary role related to our request for their participation is highlighted by shading. Their other ‘hats’ are noted by the symbol ‘√’. In total, this sample represented 59 ‘hats’ (29 being the primary reason the participant had been invited to participate in the study, and 30 additional ‘hats’). The views and experiences of this multi-faceted sample underpinned their rich insights into AH PHC CPG activities, and provided inbuilt validation of findings within, and between clusters.

**Table 1:** Participants and their roles in practice

participant	mechanism	NDoH	ProvincialDoH	academic	district/subdistrict manager	clinician public	clinician private	medical aid	prof assoc	consultant	RuReHSA	SAGE
1	interview		√		P	√					√	
2	interview		P									
3	interview	√		P								
4	interview		P									
5	interview					√				P	√	
6	interview		P								√	
7	interview		P								√	
8	interview			P								
9	interview				P	√					√	
10	interview			√			P		√			
11	interview						√		P			
12	interview			P					P			
13	interview	√		√	P					√	√	
14	interview	P										
15	interview									P		
16	interview	P										
17	interview				P	√						
18	meeting notes				P				√			
19	meeting notes		√		P	√						
20	interview		P			√					√	
21	meeting notes						√				√	P
22	meeting notes			P								√
23	interview							P				
24	interview		P									
25	interview		P									
26	interview						√		P		√	
27	interview								P		√	
28	interview			P								
29	interview			√					P			

Legend

xP primary role

√ other roles

Podiatry was significantly under-represented compared with the other three AH therapy disciplines. This reflected its presence largely only in one province, and the small number of practitioners found in private and public settings. At present, there is only one podiatry training school in South Africa, and thus the effectiveness and reach of podiatry in SA PHC is yet to be fully explored.

#### Advice

AH leaders at National and provincial government regularly sought and received guidance from AH therapy-specific forums, mostly comprising public sector clinicians, managers, and academics, and occasionally, professional associations. These forums provided conduits between policy-makers, managers and service providers for service quality to be discussed. However these forums rarely led to writing or implementing clinical guidance. The timing and membership of these fora was ad hoc*,* but the meetings provided important opportunities for policy-makers to provide to, and receive advice from, key AH players. Occasional advice was provided to these fora by private consultants who provided commissioned reports on specific issues.

#### Sectors and collaboration

In the private sector, the AH clinicians, professional associations, and university training programs tended to operate in isolation. Private insurers provided AH discipline-specific rebates for specific services, which did not foster collaborative or multidisciplinary AH evidence-based practice. However the public sector had a different approach. Whilst PHC AH services could be delivered as either single disciplines or in multidisciplinary teams, the service was generally considered to be ‘rehabilitation’, which provided a corporate ‘identity’ and collaborative focus for the AH therapies. At National and provincial government levels there were disability and rehabilitation portfolios (albeit understaffed), which fostered a multidisciplinary AH view which could resonate from top down if there were sufficient resources *‘rehab doesn't have a very high priority and it is not very valued in the Department, so there is no a lot of emphasis and technical reserves to the way that they invest and structure and develop the services’*.

We also found informal, volunteer, vibrant multidisciplinary and cross-sector groups such as Disability Action Research Team (DART), the Rural Health Advocacy Group and Rural Rehabilitation South Africa (RuReSA). Membership was from academia, professional associations, all levels of government and the private sector. These groups were faced with the needs-based immediate challenges of leading best practice service delivery, and providing AH services in underfunded PHC settings dealing with patients with huge disability need across the lifespan.

*‘Lots of questions still unanswered, like why isn’t community based rehabilitation part of a strategy in South Africa, why isn’t it funded and resourced’.*

These groups provided informal support networks and fostered the activities of individuals with interests in AH CPGs. They developed innovative ways of working with scant resources, and often developed clever guidance documents to support practitioners working in isolation in challenging clinical environments. Whilst they called them CPGs, none of the ones cited complied with international best-practice in construction or presentation [2–4].

*‘Now the CPGs that we were developing are different from the Government policy documents, we need that type of thing to include research analysis and look for clinical reasoning and evidence to come up with those clinical guidelines’*.

AH clinicians working in policy, planning and service delivery management positions in Government reported retaining their AH ‘clinical lens’ when shaping, organising and implementing services, even for conditions that did not require AH care (such as HIV and TB). These people were critical in ensuring an AH voice at the table, during funding and service delivery discussions.

*‘I do strategic stuff around PHC but because I come from a rehab background I try to ensure that those flavours always came into the work and it was also... I worked mostly with doctors so trying to sensitize them around the value of what the allied health professionals offer and how it can make a difference in terms of thinking about the bigger health system’*

AH clinicians working as managers at Department of Health Provincial district and subdistrict levels were keenly aware of the need to optimise the value and outcome of AH contacts with every patient. They were adroit at supporting service substitution (different AH clinicians substituted for each other) in the absence of providers from other AH disciplines. Community-based health promotion, chronic disease self-management and sustainable community initiatives that supported individuals to improve function once therapy sessions were completed were commonly discussed and implemented, despite lack of guidance as to how to do it, and resource constraints. Trial programs of what was considered to be ‘best practice’ service delivery were identified during discussions, although these were mostly conducted on shoe-string budgets and voluntary goodwill, and were rarely evaluated, or sustained after the initial trial period.

*‘I know I can go to colleagues and ask for help to run a pilot program, with only a bit of money from savings, and everyone just jumps in and does it, but the money and goodwill run out, and we don’t have anything sustainable’*

### Guidance

*Nomenclature:* Participants generally used the term ‘guidelines’ or CPGs in their discussions, however it became apparent during the first few interviews that this term meant different things to different people, in different circumstances and for different purposes. We often needed to physically see a ‘guideline’ before we could understand its intent, or classify it. ‘Guidelines’ could mean comprehensive, evidence-based CPGs; Government or medical aid policies and directives; practice principles or standard operating procedures/ scope of practice for managers; or clinical end-user guidance such as protocols, algorithms, decision-making tools.

*‘I don’t think there are any guidelines, CPGs that relate to rehab because I haven’t come across them. What I have come across specifically is to do with spinal cord injury, stroke and traumatic brain injury and cerebral palsy but not a national level, more at different hospitals actually or at provincial level’.*

#### Drivers

Directives for practice largely came from the National Department of Health (NDoH) in the public sector, and compensable bodies (insurers) in the private sector. These directives were sent to provincial Department of Health (DoH) or professional associations respectively, for action. When we sighted ‘guidelines’ produced by Government, they were more in the form of policy, directives, circulars or aspirational statements. They related to meeting national or provincial service delivery targets or achieving broad health outcomes (such as Millennium Development Goals), rather than the implementation of evidence-based care.

*‘one of the biggest challenges is that at National the focus is on policy issues, and provinces and districts the focus is on implementation’*

Mostly what we viewed were purpose-built decision-making tools, or directives, aimed to meeting end-user needs, few with an evidence-base. This approach reflected an urgent need to address a problem, the time available, knowledge about CPGs, and recognition of the needs of end-users.

*‘the best practice statements or protocols are developed ad hoc It’s more like recommendations. It’s not do this, do this. It’s more we have found this worked and then kind of highlighting what it is that the findings or tools were used. How the tools were used. Who used the tools and on whom the tools are being used and probably that is kind of what we share with one another but there are conversations around so I am using this and then next month so we decide okay we’re all going to try this out but next time we meet you may have found that you know what it’s not really working for me. So we try and include the variations as well but it really is very informal at this stage.’*

Another participant indicated that:

*‘there was no literature review done, there was no audit done of the provinces, what was going on in the provinces, so the document that I developed which is a lot to do with the prevention of disability and a lot to do with PHC and what needs to be done for rehab to be relevant to actually meet those goals of prevention, it can’t be done without knowing what is available and what human resources are available at the provincial level.*

#### Purpose

The purpose of CPG activities in AH PHC was unclear. Participants’ answers reflected a range of different and often complex situations for which guidance was required, who asked for guidance, where the answers could be found, and by whom, and what answers could be provided in what circumstances. Rather than summaries of best research evidence for specific tasks, guidance was mostly about service delivery (who should undertake tasks, with what training, under what circumstances, with what equipment, how often, for how long, and with what supervision). There was a broad recognition that service delivery information should be coupled with best current management evidence so that purchasers (governments, insurers) could determine the best ways to spend scarce available resources, but there was no clear understanding about how this information could be produced.

*‘You have limited resources you can’t cover all the areas that you want to cover. We have a skewed situation, you know, in terms of distribution in South Africa where our metropolitan areas … ideally you know is staffed well but when you go out into remote areas you find very limited and sometimes even no presence. So, so that’s one of the big key things. The other thing is as much as people would want to provide a comprehensive service, we, we still face, you know, challenges with providing the necessary equipment’.*

Another participant indicated that guidance were more about regulating funding and purchasing rather than clinical judgement and evidence.

*‘We do not develop anything clinical in terms of guidelines to tell practitioners how to practice, ever. All our policies and “guidelines” are directed at how we would fund’.*

#### Evidence sources

Participants acknowledged that research evidence was important. However whilst we heard about attempts to use research evidence to answer questions, we also heard about frustration with the available AH evidence base in terms of its limitations, and the need to adapt or contextualise it to local situations. Moreover, engagement of ‘experts’ (academics in particular) on Government advice panels or fora seemed to be underpinned by an assumption that they brought the research knowledge with them (rather than using independent robust evidence reviews to answer clinical questions).

*We have what is called the Provincial CPG Committees, for example we have got one around child health. So you know you have a group of clinicians that sit together and they develop CPGs, let say, for a specific health condition.*

*‘You assume that a group of experts will know all the background evidence, we don’t have time or the need to look any further for it’.*

*‘They get a group of experts which we do have in this country on various subjects who are internationally renowned …… to participate in the Advisory Board. Where (the evidence) was seen to be obvious that it was common to South Africa those parts, I think, were glossed over and we summarised those but we had specific interest which were relevant to our population’.*

### Role of AH in PHC

#### Discipline groupings

AH services were delivered in PHC settings either as single therapy disciplines or as multidisciplinary practices. In the private sector, the most common form of service delivery was single disciplines, a service approach reinforced by medical aids rebates. However in the public sector, we heard about the significant focus on rehabilitation, particularly at the coal face of PHC, the community, in the area of disability management and rehabilitation.

#### Disability and rehabilitation

Sound statistics on South African disability prevalence are lacking, although it is widely recognised that disability significantly constrains individual, community, provincial and national productivity)^47^. Participants talked about their frustration in not having the impact of disability noticed at National and Provincial government. The main reason for this was generally believed to be that disability paled into insignificance in the face of the ongoing fatal burden of diseases such as TB, HIV and childhood illness. Disability was the largely unmentioned and unnoticed burden that was carried by individuals, their families and their communities, with treatment variably provided, by an inadequate workforce, with ad hoc programs, and short-term project funding from NGOs, district and subdistrict pilot studies, and community engagement. Disability and chronic disease was reported as occurring across the lifespan, from children born with cerebral palsy, spinal deformities or foetal alcohol syndrome, through to acquired injuries from motor vehicle or workplace accidents, or violence, through to age- or disease related disability from stroke, falls or the legacy of HIV or TB. In a country where saving lives has the measure of health success [22, 25, 26, 28, 29], optimising quality of life, function and productivity has been of far less interest and value. However we heard of the significant burden of disability, and how with individual, family and community-empowerment, inexpensive AH interventions to improve strength, balance, and motor control, disabled individuals could be assisted to become productive members of their community.

*‘from the disability side there is a big thing about accessibility and, and we still need to somehow convince our architects that it’s better to incorporate in vessel design when you start off but they, they tend to think that you’re adding unnecessary expenses and we have new buildings that are, that are built now in the twenty-first century that are not universally accessible. So, so, it’s, it’s everything that is seen as an optional aspect it’s relegated to the bottom of the priority list you see and, and quality of life it is seen in that way’*.

#### AH recognition in the larger PHC sense

Consequently, at National and provincial Government levels, we heard how AH was not perceived as an important player in the quest to effectively manage the current priority PHC conditions.

*‘Allied health has little role in the important areas that we need to fund in PHC at the moment. They don’t save lives’*

Rehabilitation to attain optimum function and quality of life was universally acknowledged as essential in SA PHC, but there was also acknowledgement that the role of AH in assisting with this was poorly recognised at National Government level. This was at odds with the urgent on-the-ground need to deal with the tsunami of disability and chronic disease burden in the PHC sector, now that increasing lives are being saved from previously-fatal diseases such as HIV and TB.

*‘Do we put a value on quality of life or do we put a value on saving life? So, so, I mean, things that should not be mutually exclusive because you need both. You need to save a life but when you have saved it you need that life to lead you know a decent life after that. So, so putting a value on quality of life is tricky’*

We heard about situations where significant efforts had been made in AH working parties (comprising clinicians, academics and province managers working largely voluntarily), to develop frameworks and guidance for best practice service delivery and quality care, only to have these efforts dismissed and ‘watered down’ in policy documents, because of the lack of focus at government level on AH PHC services (now, and in the future).

*‘we put such a lot of effort into making clear statements of what we needed to make rehab work in communities, and when the policy came down, it was, like, so watered down, it meant nothing’*

At all levels of government we heard time and again of the muted AH voice in making local service delivery and resource allocation decisions. We heard stories about lack of funding for training and future workforce planning, resource constraints on AH services (not enough AH staff, not enough experienced supervision at point of contact with patients, difficulty in managing huge workloads, large waiting lists), and lack of guidance about best practice, effective care that could assist in reducing waiting lists and engaging patients in their own care. Despite this, there was a perception of hope that things would and could change. By drawing on vibrant discipline-specific and multidisciplinary AH groups which aimed for excellence, despite resource limitations, lack of guidance regarding best practice, changing political agendas and lack of recognition of the value AH in the current PHC system, the importance of a concerted, evidence-based national and provincial agenda for PHC AH activities in South Africa would be recognised.

*‘I have seen a lot of development from an era where Allied Health Rehabilitation was never part of the narrative, it wasn’t there. It didn’t appear anywhere. It didn’t appear in policy documents, it didn’t appear you know in discussion. It didn’t feature anywhere to an era where the complaint is we’re not taken too seriously enough. At least we are featuring you see. So from an era where we didn’t feature to an era where you feature but the challenge is the priority is not, is still not enough’*

### Framework

**Fig. 1:**
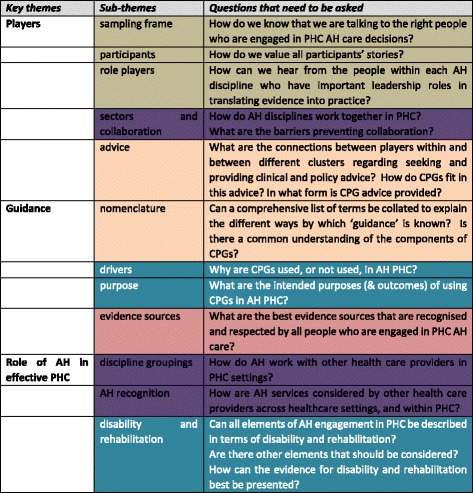
Framework to assist in determining the ‘state of play’ of uptake of AH CPGs in PHC settings. Code: The colours identify sub-themes that are linked by intent. Whilst some sub-themes were linked to different key themes, they addressed different aspects of the same issue. These issues are identified by the use of the same colour.

Determining how CPGs factored into South African AH PHC activities presented a complex methodological challenge. We needed to understand who the players were, and ask the right questions (at interview, and in analysis) to understand the complexities of the issues. This framework highlighted the non-linearity and exclusivity of the sub-themes, and how they were interlinked. This framework may efficiently assist future research in South Africa regarding AH CPG activities in PHC settings, and how CPGs could assist in PHC policy formulation from National government flowing through to local implementation activities. The framework may also assist other countries to consider CPG activities and evidence implementation without having to undertake the background work that we did, to establish comprehensive understandings of current activities. The colours in Fig. 1 identify sub-themes from our data analysis that are linked across key themes by intent. They identified different aspects of, and lenses on, the same issue.

## Discussion

This study presents comprehensive and new information on the role of CPGs in AH PHC, in a country which has significant challenges in dealing with an increasing burden of chronic disease and disability. Our cluster sampling approach was supported, as we heard of no new clusters during the interviews [33, 37, 40]. Pre-planning the clusters and asking for new participants throughout the interviews, ensured that we heard from the right people, to develop a clear understanding of the current complex ‘state of play’ of CPGs in AH engagement with South African PHC settings.

The need for evidence-based guidance for quality AH PHC practices was widely acknowledged, but significant barriers to obtaining and implementing such guidance were also identified. These barriers replicated reports from other countries [17, 18], however there were additional complexities in terms of South African PHC needs [22, 24–28]. There was a widespread belief that implementation of good quality CPGs might improve the quality of local care, and assist in dealing with scant resources, huge community need, lack of standardised training, and Government focus on priorities other than quality of life and functionality. However there was also a disconnect between participants’ perceptions of what CPGs were, how they were produced, and how guidance was presented and implemented in local PHC settings, and the realities of effectively producing and putting locally-relevant CPGs into practice across the country.

AH therapies have a remit ‘*to develop, maintain and restore maximum movement and functional ability throughout the lifespan. This includes providing services in circumstances where movement and function are threatened by ageing, injury, pain, diseases, disorders, conditions or environmental factors. Functional movement is central to what it means to be healthy’ (WCPT 2011)* [41]. This puts AH therapies at the forefront in SA PHC, in dealing proactively with the tsunami of improving the quality of lives saved from previously fatal communicable diseases.

In our framework (Fig. 1), we have provided a methodological approach that could assist researchers in South Africa, and other countries, to investigate local AH perspectives on CPGs. This framework is particularly relevant in situations where AH PHC is variably available, affordable or accessible, and where there are complexities in how AH care is delivered, funded and consumed.

We found a range of clinical guidance in SA for AH PHC, presented in many forms. Whilst it had been produced with the best of intentions, little of it was based on best evidence, none of it was peer-reviewed or available through a central repository, and most of it related to local service delivery issues that may not be generalisable. Many of the existing (so called) CPGs were not based on best practice CPG development principles [2–4], and the underpinning evidence-bases were questionable. Thus these documents could not realistically be called CPGs because they did not meet international standards [2–4]. The gaps between what is expected international best practice in CPG writing, and what was produced, was the result of lack of understanding of the elements of CPGs, coupled with lack of training, time, expertise, manpower, access to evidence sources, being clear on the purpose of the guidance, and financial resources.

Significant support exists across SA for better quality AH CPG writing and implementation, if one capitalises on the vibrant informal AH networks, and the formal organisations such as medical aids (insurers), professional associations, academic institutions and national and provincial government disability portfolios. However training is required to build capacity, ensure standard understanding, and efficiency of effort. Thus the way forward is to offer specific training, develop a central CPG repository, and seek formal supports from government, and national and local organizations in order to build knowledge, skills and capacity.

## Conclusion

The participants in this study provided a consistently-expressed desire for quality guidance to support AH PHC activities around South Africa, specifically to deal with the increasing national burden of disability and chronic disease. No international CPGs were used, and there were no South African AH CPGs. The guidance gap was filled by non-evidence-based documents produced often without training, to deal with specific clinical situations. This led to frustration, duplication and fragmentation of effort, confusing nomenclature, and an urgent need for standardised and agreed guidance. Most of the guidance documents dealt with service delivery issues rather than interventions. The situation is ripe to explore efficiencies in using guidance from evidence-based CPGs from other countries, and contextualising these to local circumstances.

## Additional file

Additional file 1: Interview Guide (DOCX 14 kb)

## Abbreviations

AH: Allied Health
COREQ: Consolidated criteria for reporting qualitative research
CPG: Clinical Practice Guidelines
DART: Disability Action Research Team
DoH: Department of Health
NDoH: National Department of Health
PHC: Primary Health Care
RuReSA: Rural Rehabilitation South Africa
SAGE: South African Guideline Evaluation
