# Supplementary material for: South African primary health care allied health clinical practice guidelines: the big picture
Source: BMC Health Serv Res. 2018 Jan 29;18:48. doi: 10.1186/s12913-018-2837-z (PMC5789688; doi:10.1186/s12913-018-2837-z)
Supplement: Additional file 1: — Interview Guide (DOCX 14 kb) [file 12913_2018_2837_MOESM1_ESM.docx]

**Additional file 1** Interview guide

1. ***Who are you?***

Ask about the demographic and institutional profiles of CPG ‘players’.

1. ***What do you think?***

Ask them what they understand by the concept of PHC in South African context. What are the key PHC conditions that this project should focus on?

Explore their views on the current processes of identifying appropriate guidelines, developing and/or contextualising guidelines, putting them into practice, educating others about them, using them to assess changes in clinical behaviours etc.

1. ***What do you require?***

Assess their role, skills and resources and what their needs are with respect to CPGs for improving practices.

1. ***What processes do you have in place?***

Identify existing frameworks, supports, activities that are being used.

1. ***What is your experience of using research?***

Assess their experience in using research to inform CPG development. Ask about their understanding and knowledge about end-user experiences regarding putting appropriate CPG recommendations into practice.

1. ***What barriers and facilitators have you considered/ experienced in implementing guidelines?***

Discuss the barriers and potential solutions, including any ‘good news’ stories for use of guidelines in PHC facilities in SA.

1. ***What context are you working in?***

Explore internal and external contexts in which guidelines are formulated and implemented, including the drivers/ motivation for these activities.

1. ***What networks exist already?***

Explore the contexts and networks within which South African CPGs are developed, implemented, used and evaluated, and who the stakeholders are in these activities
